# Supplementary material for: Experimental Evolution Reveals a Novel Ene Reductase That Detoxifies α,β-Unsaturated Aldehydes in Listeria monocytogenes
Source: Microbiol Spectr. 2023 Apr 10;11(3):e04877-22. doi: 10.1128/spectrum.04877-22 (PMC10269891; doi:10.1128/spectrum.04877-22)
Supplement: Supplemental file 1 — Supplemental material. Download spectrum.04877-22-s0001.pdf, PDF file, 0.5 MB [file spectrum.04877-22-s0001.pdf]

Supplementary material

Table S1. List of MS ions used to quantify and qualify t-CIN, ethyl benzoate, and t-CIN metabolites from the HS-SPME-GC-MS analysis in Figure 6.

Compound	Quantification (m/z)	Qualification (m/z)
trans-Cinnamaldehyde	131	103, 77
Ethyl benzoate	105	77, 122, 150
3-Phenyl-2-propenol	92	91, 134
3-Phenylpropanal	91	92, 134
3-Phenylpropanol	91	92, 117, 118

Table S2. Selection of homologs of *L. monocytogenes* YhfK retrieved by BLASTP analysis and represented in a phylogeny tree shown in Figure 9.

**Organism**	**GenBank acc.**	**E-value**	**Identity**
Listeria monocytogenes str. Scott A	EGJ25920	na	100%
Abyssicoccus albus	WP_077139846	2.0E-44	39.71%
Acinetobacter calcoaceticus DSM 30006	WP_005047009	5.0E-25	35.62%
Actinocatenispora sera	WP_051802873	5.0E-23	34.27%
Actinoplanes friuliensis DSM 7358	WP_023561358	3.0E-36	40.85%
Actinosynnema mirum DSM 43827	WP_012782666	1.0E-26	34.76%
Alcanivorax borkumensis SK2	WP_011588187	8.0E-54	47.83%
Alkalihalobacillus clausii KSM-K16	WP_035201633	1.0E-50	43.27%
Alkalihalobacillus halodurans C-125	WP_010897685	6.0E-55	49.51%
Alkalihalobacillus pseudofirmus OF4	WP_012958351	3.0E-54	45.75%
Altererythrobacter epoxidivorans	WP_061925926	3.0E-34	39.71%
Alteromonas mediterranea U8	WP_020743433	3.0E-36	38.28%
Amycolatopsis methanolica 239	WP_017986042	3.0E-27	35.21%
Arthrobacter citreus	WP_152228138	1.0E-33	36.79%
Arthrobacter crystallopoietes BAB-32	WP_005269228	9.0E-32	34.74%
Bacillus amyloliquefaciens LL3	WP_068444393	1.0E-52	44.50%
Bacillus methanolicus MGA3	WP_003349462	2.0E-50	42.03%
Bacillus subtilis 168	WP_003245344	1.0E-38	43.00%
Brochothrix thermosphacta ATCC 11509	WP_029091388	5.0E-54	45.19%
Carnobacterium inhibens K1	WP_034537835	1.0E-34	36.97%
Carnobacterium sp. 17-4	WP_013709813	3.0E-47	42.79%
Cellulomonas flavigena DSM 20109	WP_013118449	1.0E-28	35.03%
Cellulophaga baltica 18	WP_029444924	4.0E-41	40.00%

Chromohalobacter salexigens 1H11	WP_035410475	1.0E-38	37.56%
Croceibacter atlanticus HTCC2559	WP_013187612	3.0E-44	42.16%
Cytobacillus oceanisediminis	WP_019383485	4.0E-47	41.18%
Enterococcus gallinarum EG2	WP_003127925	5.0E-40	40.00%
Exiguobacterium antarcticum B7	WP_014970171	1.0E-47	42.65%
Furfurilactobacillus rossiae L1	WP_017260448	2.0E-36	37.16%
Gramella forsetii KT0803	WP_011709802	1.0E-37	40.39%
Halalkalicoccus jeotgali B3	WP_008415754	2.0E-45	42.23%
Halobacillus halophilus DSM 2266	WP_224895793	3.0E-56	47.32%
Halobacillus litoralis	WP_160916865	2.0E-58	49.28%
Halobacterium salinarum R1	WP_012289566	8.0E-49	42.44%
Haloferax gibbonsii ATCC 33959	WP_004975949	5.0E-44	40.28%
Haloferax volcanii DS2	WP_004041980	3.0E-43	43.62%
Halomonas titanicae BH1	WP_039859619	2.0E-43	39.51%
Halorhodospira halophila	WP_011813864	2.0E-42	41.26%
Halostagnicola larsenii JCM 13463	WP_049953373	3.0E-47	40.58%
Haloterrigena turkmenica DSM 5511	WP_012942372	3.0E-48	43.33%
Halovivax ruber JCM 13892	WP_015299416	2.0E-49	41.43%
Hirschia baltica DSM 5838	WP_012778037	1.0E-34	38.24%
Hoeflea sp. IMCC20628	WP_197078399	3.0E-35	41.58%
Ilumatobacter coccineus YM16-304	WP_015441337	2.0E-27	36.41%
Jeotgalibacillus malaysiensis	WP_039810704	7.0E-65	44.28%
Kytococcus sedentarius DSM 20547	WP_012801883	1.0E-34	35.55%
Lacticaseibacillus casei 12A	WP_087911616	6.0E-32	38.79%
Lactiplantibacillus plantarum ZJ316	WP_063488272	1.0E-38	39.90%
Lactococcus garvieae ATCC 49156	WP_213433223	1.0E-36	37.85%
Lactococcus lactis subsp. lactis	WP_010905761	1.0E-38	43.00%
Latilactobacillus sakei subsp. sakei	WP_094365887	3.0E-19	30.52%
Levilactobacillus brevis ATCC 367	WP_039106899	1.0E-29	37.56%
Ligilactobacillus acidipiscis DSM 15836	WP_010494836	1.0E-28	38.46%
Listeria grayi ATCC 19120	WP_036108203	1.0E-81	60.00%
Listeria innocua FSL J1-023	WP_003768174	1.0E-142	93.78%
Listeria seeligeri FSL N1-067	WP_003749357	9.0E-122	81.34%
Loigolactobacillus coryniformis subsp. torquens	WP_003678857	1.0E-32	38.71%
Maribacter hydrothermalis	WP_068484818	1.0E-43	41.63%
Marinilactibacillus sp. 15R	WP_072693299	6.0E-46	40.57%
Marinobacter nauticus ATCC 49840	WP_072676559	3.0E-59	47.60%
Marinobacterium jannaschii	WP_027859208	3.0E-41	37.98%
Marinomonas communis	WP_228189941	2.0E-40	37.56%
Metabacillus sediminilitoris	WP_136354179	1.0E-52	44.50%
Methylophaga frappieri	WP_014703251	4.0E-43	41.46%
Mycobacterium marinum M	WP_038580518	7.0E-28	36.67%

Mycolicibacterium smegmatis MC2 155	WP_011728707	3.0E-26	34.88%
Natrialba magadii DSM 3394	WP_004215569	1.0E-43	40.67%
Natrinema pallidum DSM 3751	WP_006185363	1.0E-42	40.19%
Natrinema versiforme JCM 10478	WP_049890524	8.0E-49	43.20%
Natronococcus occultus DSM 3396	WP_015320273	6.0E-51	43.60%
Nonlabens dokdonensis DSW-6	WP_015363198	2.0E-38	37.80%
Oceanobacillus iheyensis HTE831	WP_011067527	9.0E-56	45.93%
Paenibacillus polymyxa SC2	WP_013373679	2.0E-59	48.31%
Parageobacillus caldoxylosilyticus	WP_042411246	1.0E-53	48.10%
Parvularcula bermudensis	WP_013300417	2.0E-36	40.10%
Pediococcus claussenii ATCC BAA-344	WP_014214583	4.0E-36	37.98%
Peribacillus muralis	WP_057912132	1.0E-59	44.08%
Phaeobacter inhibens DSM 17395	WP_014873834	7.0E-36	41.29%
Planococcus antarcticus DSM 14505	WP_006828158	6.0E-55	47.37%
Polaribacter reichenbachii	WP_068360023	1.0E-41	41.95%
Pontibacillus sp. HMF3514	WP_160098489	7.0E-57	47.09%
Priestia megaterium DSM 319	WP_214985613	1.0E-63	50.72%
Pseudoalteromonas arctica	WP_010554646	3.0E-32	40.20%
Psychrobacillus sp. AK 1817	WP_151109713	1.0E-50	43.96%
Psychroflexus torquis ACAM 623	WP_015022789	2.0E-37	39.81%
Rathayibacter rathayi NCPPB 2980	WP_097165845	4.0E-34	35.71%
Rhodococcus erythropolis CCM2595	WP_020970330	5.0E-26	34.74%
Robiginitalea biformata HTCC2501	WP_015755446	4.0E-41	41.55%
Rubrobacter radiotolerans DSM 5868	WP_038680084	3.0E-32	40.19%
Salimicrobium jeotgali	WP_008591040	2.0E-52	46.34%
Salinicoccus halodurans	WP_046789033	6.0E-46	38.76%
Salinisphaera sp. LB1	WP_109994268	1.0E-44	41.75%
Salisediminibacterium selenitireducens	WP_013171998	6.0E-51	42.92%
Shewanella frigidimarina NCIMB 400	WP_011635783	6.0E-41	42.16%
Sphingorhabdus sp. M41	WP_067201614	3.0E-34	39.71%
Staphylococcus aureus M1	WP_001024094	3.0E-38	37.91%
Staphylococcus equorum subsp. equorum	WP_046466170	3.0E-34	38.86%
Streptococcus gallolyticus	WP_061459807	1.0E-28	35.02%
Streptomyces albulus ZPM	AKA04559	6.0E-23	33.18%
Sutcliffiella horikoshii	WP_088017909	1.0E-43	40.19%
Tateyamaria omphalii	WP_076626432	4.0E-37	41.50%
Tetragenococcus halophilus NBRC 12172	WP_014124834	2.0E-45	42.33%
Virgibacillus pantothenticus	WP_082240849	2.0E-47	41.90%
Weissella hellenica	WP_074427066	2.0E-43	40.89%

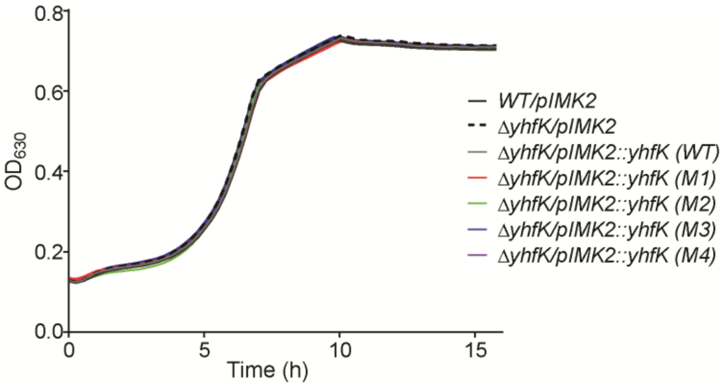

Figure S1. Growth of *L. monocytogenes* Δ*yhfK* complemented with WT or mutated *yhfK* alleles at 30ºC in BHI. The data represent the average of measurements of three independent cultures. The SD is omitted for clarity.

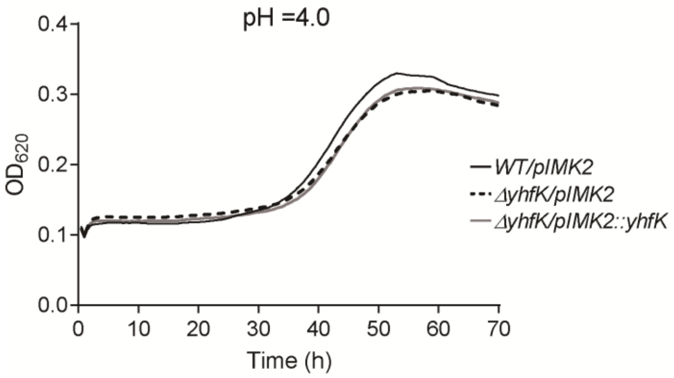

Figure S2. Growth of *L. monocytogenes* strains at 30ºC in BHI at pH = 4.0. The data represent the average of measurements of three independent cultures. The standard deviation is omitted for clarity.

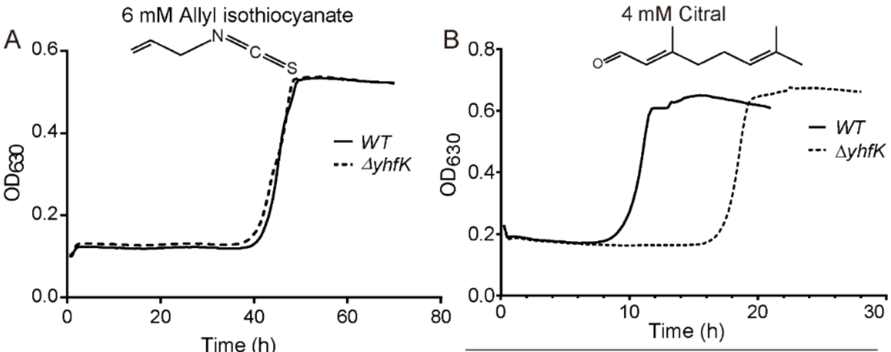

Strain	*WT*	*ΔyhfK*
lag phase (h)	8.3 ± 1.4^{A}	17.6 ± 0.6^{B}
ODmax	0.65 ± 0.00^{A}	0.66 ± 0.01^{A}
μmax (/h)	0.073 ± 0.008^{A}	0.060 ± 0.006^{A}

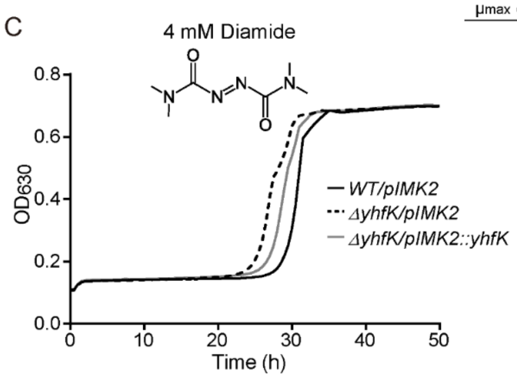

Figure S3. Growth of *L. monocytogenes* in BHI supplemented with 6 mM allyl isothiocyanate (**A**), 4 mM citral (**B**), and 4 mM diamide (**C**). All growth curves represent the average of measurements of three independent cultures, but the standard deviation is omitted for clarity. The growth parameters (λ, μmax, and ODmax) are represented in the table as mean ± SD; n = 3. Values followed by a common letter are not significantly different at the 5% level. The molecular structures of allyl isothiocyanate, citral (E-isomer), and diamide are shown.

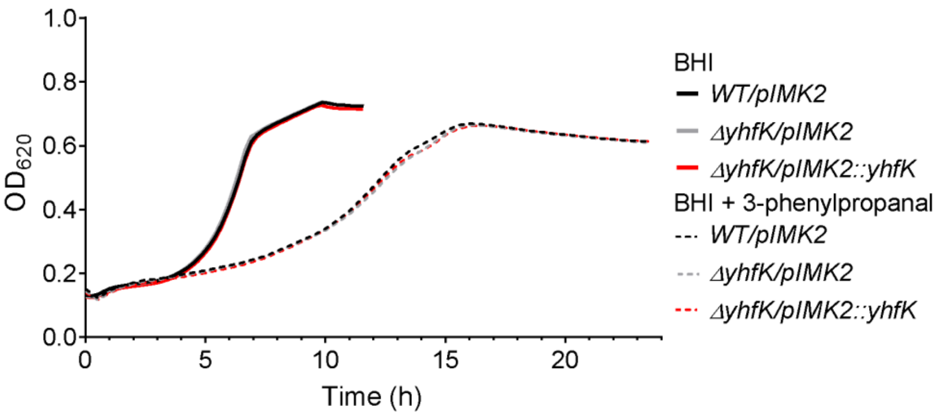

Figure S4. The growth of *L. monocytogenes WT/pIMK2*, Δ*yhfK/pIMK2*, and Δ*yhfK/pIMK2::yhfK* in BHI and BHI supplemented with 4 mM 3-phenylpropanal. The curve represents the average of measurements of three independent cultures. The standard deviation is omitted for clarity.
